# The chemfp project

**DOI:** 10.1186/s13321-019-0398-8

**Published:** 2019-12-05

**Authors:** Andrew Dalke

**Affiliations:** Andrew Dalke Scientific AB, Trollhättan, Sweden

**Keywords:** Molecular fingerprints, Similarity searching, Tanimoto, High-performance, Format, Open source, FOSS, Performance benchmark

## Abstract

The chemfp project has had four main goals: (1) promote the FPS format as a text-based exchange format for dense binary cheminformatics fingerprints, (2) develop a high-performance implementation of the BitBound algorithm that could be used as an effective baseline to benchmark new similarity search implementations, (3) experiment with funding a pure open source software project through commercial sales, and (4) publish the results and lessons learned as a guide for future implementors. The FPS format has had only minor success, though it did influence development of the FPB binary format, which is faster to load but more complex. Both are summarized. The chemfp benchmark and the no-cost/open source version of chemfp are proposed as a reference baseline to evaluate the effectiveness of other similarity search tools. They are used to evaluate the faster commercial version of chemfp, which can test 130 million 1024-bit fingerprint Tanimotos per second on a single core of a standard x86-64 server machine. When combined with the BitBound algorithm, a k = 1000 nearest-neighbor search of the 1.8 million 2048-bit Morgan fingerprints of ChEMBL 24 averages 27 ms/query. The same search of 970 million PubChem fingerprints averages 220 ms/query, making chemfp one of the fastest CPU-based similarity search implementations. Modern CPUs are fast enough that memory bandwidth and latency are now important factors. Single-threaded search uses most of the available memory bandwidth. Sorting the fingerprints by popcount improves memory coherency, which when combined with 4 OpenMP threads makes it possible to construct an N × N similarity matrix for 1 million fingerprints in about 30 min. These observations may affect the interpretation of previous publications which assumed that search was strongly CPU bound. The chemfp project funding came from selling a purely open-source software product. Several product business models were tried, but none proved sustainable. Some of the experiences are discussed, in order to contribute to the ongoing conversation on the role of open source software in cheminformatics.
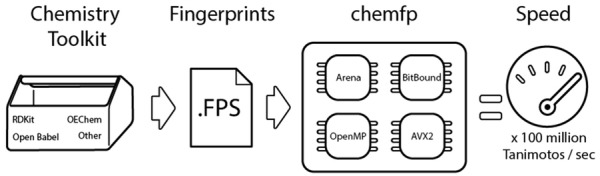

## Introduction

Molecular similarity search is a fundamental concept in cheminformatics. The most common form is almost certainly a Tanimoto similarity search of bitstring fingerprints. Complete search systems are available from many vendors, or a good programmer can implement a basic system with reasonable search performance in only a few hours. High-performance search systems, which combine fast popcount evaluation and pruning algorithms, require significantly more development effort. This paper starts with a review of those approaches, many of which are either described in the cheminformatics literature in an incrementalist fashion which make them difficult to discover, or only published in the specialist literature of other fields.

The chemfp project started in order to develop a de facto file format for chemical fingerprints. This requires some consideration of why such a format did not already exist, in order to understand which factors to focus on during format development. Two formats were developed; the text-based FPS exchange format, which is simple to read and write, easily compressed, and appropriate for streaming workflows, and the binary FPB application format which is more complex and requires random-access reads, but has significantly shorter load times.

The chemfp package for Python includes optimized threshold and *k*-nearest implementations FPS file scan search implementations, highly-optimized implementations of the BitBound pruning method to search data sets either loaded into memory or memory-mapped from an FPB file, and OpenMP parallelized BitBound methods for N × M and N × M similarity matrix generation. The file formats and search performance are evaluated using datasets from ChEMBL and PubChem, with fingerprints generated by the Open Babel, RDKit, OpenEye, and CACTVS toolkits. While these datasets were not designed for direct scientific utility, it is hoped that they, along with a set of common search tasks, may be a common benchmark for similarity search performance. Preliminary results suggest that two of the datasets may be too sparse to provide useful comparisons between dense and sparse search algorithms.

One of the key results is that modern CPUs are extremely fast. The popcount intersection calculation, which was the limiting factor on older hardware, now requires only a few nanoseconds. This is significantly faster than the memory latency time for reading from main memory, which means that memory access issues like cache coherency have become an important limiting factor. For example, sorting the queries by popcount increases multiquery search scalability, likely because the search threads have better temporal locality. Most previous work on improved pruning methods did not consider these factors. The machine models used in older publications are discussed, along with the reasons for why their conclusions may need to be re-assessed.

Floating-point precision appears to be a subtle though common source of errors in similarity search systems, even for software written by expert developers. Examples are given as advice to future implementors, along with recommendations for how to avoid floating point calculations.

Free and open source software (“FOSS”) is popular, in part because it is typically available at no cost as the funding often comes either from volunteer contributions or indirect research funding. This paper discusses the growing understanding that this sort of funding model often has long-term sustainability problems. The chemfp project experimented with several approaches for funding the project by selling commercial software under a FOSS license. These approaches are discussed, as well as the conflicts between the economic requirements of FOSS software development and the expectations of customers used to proprietary software licensing. Chemfp is now available with cheaper proprietary licensing options as the pure FOSS funding model does not appear viable. Other funding models which may be more viable for future projects are discussed.

## Background

A fingerprint for chemical similarity is a description of a molecule such that the similarity between two descriptions give some idea of the similarity between two molecules. Willett [1], influenced by earlier work [2] showed how the Tanimoto similarity between two bitstring fingerprints is a useful mechanism to characterize molecular similarity. The Tanimoto similarity is identical to the older Jaccard similarity. The continued use of the term reflects the impact of that early work at Sheffield. The term “fingerprint” first appeared in the literature in 1992 [3] to distinguish the then-new enumeration-based Daylight fingerprints from the older substructure dictionary approach.

The late 1980s and 1990s brought an incredible growth of research as people explored ways to generate, compare, and cluster fingerprints, to extend the concept to sparse and count fingerprints, and to extend fingerprints beyond 2D substructures [4].

The most widely used fingerprints are variants of the 166-bit MACCS keys [5], Daylight linear fingerprints [6], ECFP circular fingerprints [7], and the 881-bit PubChem/CACTVS keys [8]. These are fixed-length binary fingerprints, typically with 166, 881, 1024, or 2048 bits, and with a sufficiently high bit density that they are most efficiently represented as an uncompressed bitstring instead of sparse encoding methods like an inverted index. Implementations of these fingerprints are available from a large number of tools [9].

While there are a many ways to compare two fingerprints, the vast majority use the Tanimoto similarity:

1 $$Tanimoto\left( {fp1,fp2} \right) = {{\left| {fp1 \cap fp2} \right|} \mathord{\left/ {\vphantom {{\left| {fp1 \cap fp2} \right|} {\left| {fp1 \cup fp2} \right|}}} \right. \kern-0pt} {\left| {fp1 \cup fp2} \right|}}$$

often simply referred to as “the Tanimoto”.

For binary fingerprints represented as bit strings the Tanimoto calculation can be expressed as:

2 $$popcount{{\left( {fp1\;\& \;fp2} \right)} \mathord{\left/ {\vphantom {{\left( {fp1\;\& \;fp2} \right)} {popcount\left( {fp1|fp2} \right)}}} \right. \kern-0pt} {popcount\left( {fp1|fp2} \right)}}$$

where “&” and “|” denote bitwise binary-and and -or and “*popcount()*” is the number of 1 bits in the resulting subexpressions, often called the “population count”. In chemfp, two fingerprints with 0 bits set have a Tanimoto of 0, while some other toolkits have different behaviors.

Similarity search performance can be critical. Humans typically regard response times of under 0.1 s as “instantaneous”, and start to lose focus if a search takes more a few seconds [10]. Fast search times are important not only for user-directed similarity queries, but also for secondary queries. For example, a web interface for a compound database might display a page for each compound record along with links to its 10 most similar neighbors in the database. This information can be pre-computed, which typically requires some infrastructure to compute and update the neighbor lists when the database has changed enough. On the other hand, if the search finishes within the response time budget then it can be done on-demand, which may simplify the system design.

Many algorithms are built on top of similarity search, and often the overall algorithm performance depends on the similarity search performance. For example, most of the time for the Taylor–Butina clustering algorithm [11, 12] is spent computing a sparse similarity matrix. The matrix computation is quadratic in the number of fingerprints, so a fourfold performance improvement makes it possible to work with a data set which is twice as large in the same amount of time. Performance improvements may also allow more efficient system architectures; a tenfold improvement may be fast enough that a task which required a compute cluster can now be run on a single machine, with additional savings from reducing the overhead for task partitioning and network communication.

Approximate methods for search [13] and clustering [14] with controllable error levels and better theoretical scalability for large data sets are feasible alternatives when the minimum required similarity is high enough. This paper focuses on exact methods for binary fingerprints.

## Faster Tanimoto calculations

Many people have developed techniques to improve Tanimoto similarity search performance for dense fingerprints. While many of these techniques are well-known, they have not been described in one place in the literature, and some previous papers describe inefficient implementations.

One approach is to use faster hardware and multiple cores or processors [15], or use specialized hardware like GPUs [16]. This paper focuses on x86-64 CPUs, though many of the techniques are portable to other architectures.

If there will be multiple queries against a set of target fingerprints then one often-used approach precomputes the popcount of each target fingerprint. If the query popcount is *A* and the target popcount is *B* then Eq. (2) can re-written with only the intersection popcount:

3 $$c = popcount\left( {fp1\;\& \;fp2} \right)$$

$$Tanimoto = {c \mathord{\left/ {\vphantom {c {\left( {A + B - c} \right)}}} \right. \kern-0pt} {\left( {A + B - c} \right)}} \,$$

which requires only one popcount evaluation per comparison instead of two.

Another approach is to improve the Tanimoto calculation performance through more efficient use of the hardware. Many search implementations interpret Eq. 1 literally, and represent fingerprints using a set data type and compute the Tanimoto using set operations. This approach often uses a large number of temporary set instances. By comparison, an implementation which represents a fingerprint as a byte string or sequence of machine words uses less memory, has less memory management overhead, and can implement Eq. 2 with a handful of fast bit and arithmetic operations.

Many popcount algorithms have been developed over the last 70 years [17]. Table 1 compares the relative performance of several implementations. Full details are in Additional file 1: Table S1.

**Table 1:** Relative performance of different popcount implementations

Popcount method	Performance relative to 8-bit lookup table			
	166 bits	881 bits	1024 bits	2048 bits
8-bit lookup table	1×	1×	1×	1×
16-bit lookup table	2.0	2.8	2.9	2.4
Gillies-Miller [18]	1.6	2.9	3.1	3.4
Lauradoux [19]		3.1	3.3	3.7
SSSE3 [15]			5.4	6.1
POPCNT (8 bytes/loop)				
Dispatch	3.6	6.0	6.3	6.4
Inline	4.9	6.6	6.9	6.6
POPCNT (fully unrolled)				
Dispatch	5.3	7.9	8.2	7.8
Inline	6.7	8.2	8.4	8.0
AVX2 [20] (fully unrolled)				
Dispatch			8.6	9.2
Dispatch, prefetch			8.7	9.3
Inline			9.8	9.9
Inline, prefetch			11.0	10.6

Times are scaled relative to an 8-bit lookup table, as measured by the threshold searches from the chemfp benchmark suite. In most cases the search algorithm uses a function pointer to dispatch to the appropriate popcount function, without memory prefetching. The “fully unrolled” variants implement the fingerprint popcount without using a loop. The “inline” and “prefetch” variants inline the calculation and use memory prefetching, respectively. Timings were made with chemfp 3.3. Chemfp 1.5 does not support inlining, AVX2, or prefetching

Chemfp 1.0 used a lookup table, which may be effective if table lookup is fast, but modern hardware has special methods which are faster than accessing table data even from L2 cache. Fingerprints are typically many machine words in length, so fingerprint popcounts can be computed either by summing the popcount of each word or extending a tree-of-adders approach to work on multiple words [17, 19].

While these algorithms can be implemented in standard, portable C code, faster implementations use processor-specific hardware instructions. Effectively all modern x84-64 hardware supports the POPCNT instruction, which can compute the popcount of a 64-bit word in one machine cycle. Other CPU-specific techniques were available for older consumer hardware [15]. Perhaps surprisingly, popcount implementations with AVX2 instructions outperform POPCNT-based implementations for 1024 and 2048 bit fingerprints, because the AVX2 implementation of the Harley-Seal algorithm can fetch and use 256 bits at a time while making more effective use of instruction parallelism [20]. (The POPCNT instruction on most x84-64 chips is limited to a single execution port). The VPOPCNTDQ instruction in the AVX-512 instruction set computes a 512-bit popcount, which should be faster still.

The fingerprint intersection popcount calculation is often decomposed into the sum of multiple word intersections. One optimization is to observe that many of the query words in relatively sparse fingerprints only contain 0 s. Its intersection popcount will always be 0 so does not need to be evaluated. A query-specific optimizer may also be able to merge multiple word evaluations into a single popcount, and replace some word popcounts with simple boolean expressions [21].

Other factors become important once the popcount performance is fast enough. Equation 3 requires an addition, subtraction, and division. If all targets with the same popcount are grouped together then the *A* + *B* term is constant while processing that group, removing the need for an addition.

Division is a relatively expensive operation, and might be replaced with a small lookup table [21] if table lookup is fast enough, or rewritten to use rationals and integer operations. For example, the threshold test *c*/(*A* + *B* − *c*) ≥ 0.75 may be rewritten as *c* * 4 ≥ 3 * (*A* + *B* − *c*). A further refinement for grouped fingerprints is to replace the division test with comparison to the minimum required popcount threshold shown in Eq. 4:

4 $$popcount\left( {fp1\;\& \;fp2} \right) \ge \left\lceil {{{T\left( {A + B} \right)} \mathord{\left/ {\vphantom {{T\left( {A + B} \right)} {\left( {1 + T} \right)}}} \right. \kern-0pt} {\left( {1 + T} \right)}}} \right\rceil$$

This can be calculated once for each group, which reduces the threshold test to a simple integer comparison.

On some processors, particularly older ones, misaligned data may be significantly slower or cause the program to crash, so should be memory-aligned using zero padding.

Certain fingerprint lengths are particularly common, and specialized intersection popcount functions can be written for each one with a fallback to a general purpose implementation. A fully unrolled intersection popcount for the 166-bit MACCS, assuming zero padded 64-bit words and POPCNT instruction, requires at most 12 assembly instructions and is about 40% faster than a generic loop summing the popcount of the 3 words.

The entire search algorithm can also be specialized for the most important fingerprints sizes. A threshold search for 166-bit fingerprints which inlines the intersection popcount instead of calling a function pointer is about 25% faster because it has no function call overhead and because the compiler has more ability to optimize the code. A fully-inline AVX2 search algorithm may also initialize some of the AVX2 registers once, rather than once for each intersection popcount.

The “roofline model” [22] highlights how memory latency and bandwidth become limiting factors once the popcount performance is fast enough. The absolute minimum time for a full linear search of 1 million uncompressed 1024 bit fingerprints on a machine with 20 GiB/s memory bandwidth is only 6 ms. This would require about 2.7 billion 64-bit POPCNT instructions per second, plus the operations to evaluate the Tanimoto, which is not quite achievable on a 3 GHz processor without a high degree of instruction parallelism. In practice, memory latency limitations occur before reaching bandwidth limitation, so faster AVX2 and VPOPCNTDQ implementations must use prefetching to reduce this overhead.

Finally, a *k*-nearest search may use a heap algorithm with *O*(*n log k*) performance. Tanimoto scores are ratios of two small numbers, bounded by the number of bits in the fingerprint, resulting in relatively few distinct values. For large values of *k* a counting sort [21] may be used to eliminate the *O*(*log k*) overhead.

While each optimization may only add a small performance improvements, the overall effect is multiplicative.

## Pruning methods

The fastest calculations are those which don’t need to be done. Duplicate fingerprints may be merged into a single record, which can give an appreciable speedup, especially for *O*(*n*^2^) tasks like building a similarity matrix.

Many search tools use BitBound [23] to reject obvious mismatches. If the goal is to find all target fingerprints which are at least *t* > *0* similar to a query fingerprint with a popcount of *A*, then the target fingerprint must have a popcount *B* between *B* * *t* and *B*/*t*. A value of *B* may be stored for each fingerprint, or the fingerprints may be organized into bins such that all fingerprints with the same popcount are in the same bin. The latter requires less memory storage and fewer memory accesses. The bounds give linear speedup in threshold searches, with tighter bounds as the similarity increases, and sub-linear speedup for *k*-nearest neighbors. Empirical testing using the chemfp benchmark data sets confirms that k = 1 nearest neighbor searches of MACCS and FP2 fingerprints scales as O(n^~0.65^) and the PubChem/CACTVS and Morgan searches scale as *O*(*n*^~0.8^) in the number of fingerprints in the data set (see Additional file 1: Figure S1).

Additional pruning methods include sharper M = 2 bounds [24], xor signatures [25], recursive application of the bounds to fingerprint subsets [26–28], trees [27, 29, 30] and reference points [31, 32]. These papers often demonstrate a mathematical reduction in the number of popcount operations needed, and empirically measure performance improvements over BitBound.

The best improvements occur for high similarities (typically 0.8 or above), while the overall reported performance is sometimes worse than BitBound for similarity thresholds which are both chemically reasonable and commonly used. It seems impossible to improve upon linear search for an exact similarity search of high dimensional space when using a low similarity threshold.

## Need for a fingerprint format

The chemfp project started in part to promote the FPS format as the common format for exchanging fingerprint data. Many software packages are available and in wide distribution for working with fingerprint data [9]. These in turn represent a small fraction of the fingerprint software in use, which includes personal research software and in-house tools. Yet very few tools from different origins are able to work together without some format conversion.

This should be unexpected as the beneficial network effect of an interoperable format generally causes a field to converge on one or a small number of formats in far less than the 30 years of active research on fingerprints. Nearly every tool which works with small molecules supports the SDF or SMILES file format, just like nearly every sequence analysis tools supports the FASTA format.

The success of those three formats was in no small part based on the success of respectively the MACCS II [33], Daylight [34], and FASTA [35] software, so it was clear that providing a fast similarity search tool, along with fingerprint generation tools, would help promote the FPS format. It was also clear that fast similarity search tools already existed, without resulting in a common format.

This lead to the question “Why not?”, with the hope that by identifying the factors which weaken the network effect might help improve the chances that a new format would be successful. Foremost, of course, is that most people do not need a fingerprint file format because they work with fingerprints through a database, typically via a chemically-aware database extension.

In general there are two types of fingerprint file formats: text and binary. Researchers tend to create text formats because they are easy to read and write, and to inspect visually. These formats are so simple that it is often faster for the researcher to create a new format and its I/O routines than to find if an appropriate format exists and understand someone else’s software library. It’s also typically easy—a matter of minutes—to write a converter from one format to another.

People with more advanced experience in software development tend to store fingerprints in a binary format, since binary formats are generally faster to read and write than text formats. These are also the sorts of people who write software libraries for both in-house and more general use. These binary formats are typically considered an implementation detail and subject to change as needs change. Instead, file access is mediated through command-line programs, or a library API with I/O and search routines.

What happens if a researcher wants to evaluate a new clustering algorithm implemented in R, when the fingerprint package library API is only available in C++? While R has good support for C++ bindings, it’s more likely people will write a C++ program to export the fingerprints in a new format and an R function to read that format.

Another aspect of fingerprint software is that there isn’t that much need for interoperability because essentially all of the widely-used packages as well as most in-house packages support the most common needs: fingerprint generation using variants of the MACCS, Daylight, or ECFP fingerprint types, Tanimoto similarity search for *k*-nearest neighbors and for finding all neighbors at or above a given threshold, and the *N* × *M* and *N* × *N* (symmetric) variants used for clustering and diversity selection algorithms. Most people exchange structures and treat fingerprints as derived data, to be computed when needed.

What about when there is a need? Consider a project to evaluate the relative effectiveness of circular fingerprints from different vendors. Which tool should be used for the evaluation? A surprising number of available packages and associated formats are not designed for interoperability, and cannot easily be used for this task. As two examples, the package may generate fingerprints automatically given a structure file or molecule object, but lack a way to accept an externally generated fingerprint value, or the file format may store fingerprint type parameters but not have an easy mechanism to handle foreign fingerprint types.

These considerations resulted in the working hypothesis that any format could not become a de facto exchange format unless (1) it was a text format that was as easy to read and write as the ones that researchers are used to, (2) it could demonstrate support for diverse fingerprint types, and (3) it came with a set of tools and library API which could handle most of what people needed from a fingerprint toolkit.

## Methods

### Hardware

All timings in this paper were made on a machine with a 3700 MHz i7-4770 CPU. Each core has 32 KiB each of L1d and L1i cache and 256 KiB of L2 cache. The four physical cores share 8 MiB of shared L3 cache and 32 GiB of RAM (DDR3-1600 with double channel). The single channel theoretical peak transfer rate is 12,800 MiB/s or 12.5 GiB/s. Measurements with pmbw 0.6 [36] using a single thread show a 54 GiB/s read bandwidth from L1, 35 GiB/s, from L2, 30 GiB/s from L3, and 13.3 GiB/s from RAM. The measured latencies are 1.1 ns from L1, 2.7 ns from L2, 9.6 ns from L3, and 87 ns from RAM. Chemfp was compiled with gcc 5.5.0.

### Data sets

The fingerprint data sets used in this paper are the 2048-bit RDKit Morgan fingerprints distributed as part of ChEMBL 24 [37], the 881-bit PubChem [38] fingerprints extracted from the PUBCHEM_CACTVS_SUBSKEYS tag of a PubChem mirror from 2018-12-07, and the four data sets in the chemfp benchmark. The latter contain four ~ 1 million fingerprint subsets. Three were generated from ChEMBL 23 using respectively the 166-bit OpenEye MACCS implementation, the 1021-bit Open Babel FP2 implementation, and the 2048-bit RDKit Morgan fingerprint with radius 2. The fourth contains the 881-bit PubChem fingerprints extracted from a mirror made on 2017-07-12. Table 2 summarizes the content of the search target data sets.

**Table 2:** Fingerprint target data set sizes in FPS format

Data set	#Bits	Fingerprint type	#Fingerprints (in millions)	Unique	FPS size (in MiB)	FPS.gz size (in MiB)
chemfp benchmark ChEMBL 23 subset	166	OpenEye MACCS	1.00	83.6%	54	17.7
chemfp benchmark PubChem subset	881	PubChem/CACTVS	1.00	98.2	222	53.1
chemfp benchmark ChEMBL 23 subset	1021	Open Babel FP2	1.00	96.0	258	80.5
chemfp benchmark ChEMBL 23 subset	2048	RDKit Morgan	1.00	90.6	502	59.9
ChEMBL 24	2048	RDKit Morgan	1.82	94.1	914	99.7
PubChem	881	PubChem/CACTVS	96.9	65.3	21,500	2910

“Unique” is the number of distinct fingerprints as a percentage of the total number of fingerprints

These fingerprint types were chosen because they are relatively popular and well-understood, and to give representation from each of the underlying toolkits that chemfp supports. They were not selected for any specific scientific appropriateness, and should only be used for timing purposes.

### FPS format

**Fig. 1:**
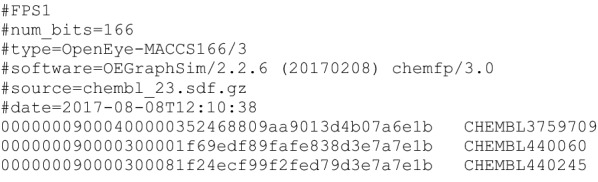
Example FPS file for 166-bit MACCS keys generated by OpenEye’s GraphSim toolkit. Header lines start with a ‘#’. The three record lines start with a hex-encoded fingerprint, followed by a tab and the record id

Figure 1 shows an example of the FPS format. It is a line-oriented text format containing an optional header section followed by zero or more fingerprint records. The header contains an optional version line, followed by zero or more metadata lines. Each header line starts with a ‘#’. Each fingerprint record contains two or more tab-separated fields. The first is the hex-encoded fingerprint and the second is the record id. The remaining fields are unspecified and may be used to store a SMILES string, activity, or other values, though the chemfp toolkit does not yet support these fields.

**Fig. 2:**
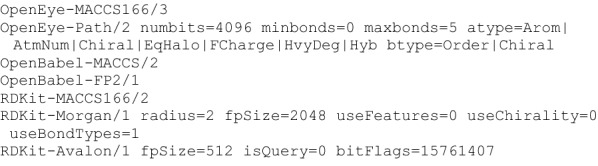
Seven fingerprint type strings from different toolkits. Each type string contains space separated terms. The first term contains the fingerprint family name and version. Remaining terms encode fingerprint parameters as key = value pairs. The OpenEye-Path and RDKit-Morgan types are wrapped over two lines for presentation

The most important metadata line, though optional, is “*type*”. It describes how the fingerprints were generated. While it can be an arbitrary text string, it should follow the format of the examples shown in Fig. 2, with one or more terms separated by a single space. The first term contains the family name and optional version. Any remaining terms are *key* = *value* pairs describing the specific fingerprint generation parameters. By convention the family name uses a prefix to indicate the tool used to generate the fingerprints, which helps distinguish between, for example, the MACCS implementations from different vendors.

As a concrete example, the second line of Fig. 2 is a type string for OpenEye’s “Path” fingerprints, version 2. It can be generated by calling OEMakePathFP() with the arguments *numbits* = 4096, *minbonds* = 0, etc. The *atype* and *btype* values describe which atom and bond properties are encoded in the fingerprint. The specific syntax is derived from the strings returned from *OEGetFPAtomType()* and *OEGetFPBondType()*.

The type has several purposes. First, it records how the fingerprints were generated. It is all too easy to create a data set then come back to it a few months later and forget how it was generated. Second, it should be machine parseable so that software can generate new fingerprints of the same type. This might be used in a search tool to figure out how to convert a new query structure into a compatible query fingerprint. Third, it should be in canonical form, such that the type strings match if and only if they describe the same fingerprint generation options. This makes it possible for even simple tools to detect if two data sets may have incompatible types.

The optional “*software*”, “*source*”, and “*date*” lines are primarily for data provenance. The software line records version information for the key fingerprint generation components. Each source line stores a filename or other description of the input to the fingerprint generation. The date line stores an ISO datetime stamp of when the fingerprints were generated.

The optional “*num_bits*” metadata line records the fingerprint length. If not present then the fingerprint length is calculated from the number of bytes in the fingerprints. Fingerprint types which are not a multiple of 8 bits long, like the 166-bit MACCS keys and the 881-bit PubChem/CACTVS fingerprints, must pad the highest bits with zeros, and should record the actual size in the *num_bits* field. The value may be used to compute length-dependent similarity coefficients, or to determine which bits are appropriate for machine learning.

The hex-encoded fingerprint is always a multiple of 2 characters in length. All fingerprints must have the same length, and 0-length fingerprints are not allowed. The bytes are ordered so bit 0 is the first bit of the first byte, that is, the bytes are in little-endian order. Examples of hex-encoded 16-bit fingerprints are: “0100” (bit 0 is set), “2000” (bit 5 is set), and “c218” (bits 1, 6, 7, 11, and 12 are set). Note that the two hex characters for each byte are in big-endian order so the hex representation nibbles are in the order “1032”.

Hex encoding was chosen because the primary goal was to make a format which was easy for most researchers to read and write with a few minutes of work. Hex encoding is easier to understand and implement than more compact encodings like Base64 and ASCII85, and most widely used programming languages have built-in support for converting between a byte string and its hex representation. In any case, gzip compression recovers most of the space overhead. Table 2 shows the compressed and uncompressed sizes of the data sets used in this paper.

The tab character is used as the delimiter because IUPAC names and even some corporate ids may contain a space, comma, or other printable character.

The format may be extended by adding new metadata lines so long as they can be ignored without affecting how to interpret an FPS file. For example, a “comment” line might store some extra information about how the fingerprints were generated, and a “stats-24” line might store statistics for bit 24. A future version of the specification will likely include a way to provide header names for the additional fields of a fingerprint record.

### FPB format

The time to parse an FPS file is quite large compared to the in-memory search time. Sometimes the load time adds too much overhead. Consider a web developer following the standard edit/reload cycle to create an application which uses several multi-million fingerprint data sets. Each reload, which is normally a fraction of a second, may take around 10 s as the fingerprints are reloaded. The load time can be deferred until first use, but that will still add noticeable time to the iterative development process.

Chemfp 2.0 added support for the FPB format, which is a more complex binary representation of a fingerprint arena that is quicker to load while still supporting the optimizations for fast similarity search. The structure is a variant of the “FourCC” file format. The file starts with an 8 byte signature followed by a series of chunks. Each chunk contains an 8 byte length field, followed by a four byte chunk type name, followed by type-specific data.

There are six defined chunk types. The META chunk contains the metadata lines from the header of the FPS format, including the leading ‘#’ and newlines. Processing ends with the FEND chunk.

The AREN, POPC, FPID, and HASH chunks contain the four distinct parts of a fingerprint arena, which will be described in a later section. The AREN chunk stores the fingerprints as a contiguous block, along with information about the fingerprint length and storage size. It also contains an initial spacer to allow the first fingerprint to be word or cache-line aligned if the file is memory-mapped. The POPC chunk stores the popcount indices into the AREN chunk for sorted arenas.

The FPID chunk stores the record identifiers as a sequence of UTF-8 encoded strings, along with an offset table to look up an identifier given its index. The HASH chunk contains a modified form of the cdb hash table [39], where the values are indices into the FPID chunk. Table 3 shows the total size and size of the largest chunks for the data files in this paper.

**Table 3:** Fingerprint data set sizes in FPB format and largest chunk sizes

Data set	#Bits	#Fingerprints (in millions)	FPB size (in MiB)	AREN size (in MiB)	FPID size (in MiB)	HASH size (in MiB)
chemfp benchmark	166	1.00	54.0	22.9	15.9	15.3
chemfp benchmark	881	1.00	134	107	11.6	15.3
chemfp benchmark	1021	1.00	153	122	15.9	15.3
chemfp benchmark	2048	1.00	275	244	15.9	15.3
ChEMBL 24	2048	1.82	501	444	29.9	27.8
PubChem	881	96.9	13,000	10,300	1130	1480

The AREN chunk contains the fingerprints, the FPID chunk contains record identifiers indexed by position, and the HASH chunk contains a hash table mapping identifiers to index

The load time is significantly shorter because the loader only needs to read enough data to identify which chunks exist and extract basic information like the metadata and fingerprint sizes.

The combination of popcount indexed arenas, BitBound, and memory-mapping work well together. For example, a command-line tool which finds all matches with at least 0.9 similarity to a given fingerprint can limit file access to only the most relevant fingerprints, and since the linear access pattern is easy to predict, the file system can prefetch the data. Tests show that most of the overall time for these sorts of simple tools is spent waiting for Python to start, even for multi-million fingerprint data sets.

Memory-mapped files can also be useful when multiple components use the same FPB file because the different components may share one copy of the static, read-only memory.

The biggest negative to using the FPB format directly, instead of a fully in-memory representation, is the relative slowness of working with identifiers. It may take several essentially random-access disk reads to get the id for a given index, which is particularly slow on hard disks. Hash table lookups may require several index lookups and so be even slower. In addition, the FPB hash table is not as optimized as the Python hash table, and each identifier lookup creates a new Python string object instead of reusing a previously loaded one. This negative is usually only noticeable when a large number of identifiers are returned.

The FPB format is designed for fingerprint data sets with a few million records, which is typical for most corporate compound collections. It has been tested with the ~ 100 million fingerprints in PubChem, though design consequences of the 32-bit hash table sets an upper limit of slightly more than 250 million fingerprints. The usual way to create an FPB file is to load a fingerprint data set into memory then save the result in FPB format. This does not work for very large data sets; the loader needs more than 30 GiB of RAM for the intermediate data structures to load PubChem. Instead, chemfp’s FPB writer supports an option to write partial information to the filesystem, typically as smaller FPB files, which are collated to create the final file. The ~ 10 GiB PubChem FPB file can be created on a machine with only a few GiB of memory, and with reasonable performance.

The FPB format allows extensions. New chunks may be added so long as they don’t break compatibility. For example, the sharper M = 2 bounds might be supported by adding a secondary sort to the AREN fingerprints based on the popcount of the odd bits for all fingerprints with the same popcount, and storing the M = 2 indices for the secondary sort in a new chunk.

While the FPS format supports additional fields for each column, there is currently no way to store that information in an FPB file.

### chemfp package

Essentially no one will use a fingerprint format simply because a specification exists. The chemfp Python package attempts to overcome the chicken-and-egg problem by distributing a Python library and a set of command-line tools for working with FPS files.

The command-line tools oe2fps, rdkit2fps and ob2fps use respectively OpenEye’s OEChem and GraphSim toolkits [40], the RDKit toolkit [41], and the Open Babel toolkit [42], to parse structure files or records and generate fingerprints. Chemfp also adds a mostly complete PubChem-like fingerprint generation implementation for each toolkit.

The sdf2fps tool extracts record identifiers and pre-computed fingerprints from tags in an SD file. For example, the “--pubchem” option extracts the id from the title line and the fingerprint from the PUBCHEM_CACTVS_SUBSKEYS tag of a PubChem file. Other supported encodings include hex, Base64, sequences of ‘0’ and ‘1’ characters in different bit orders, and the fingerprint encoding used in Daylight Thor Data Trees (TDTs).

The simsearch tool implements *k*-nearest and threshold searches of an FPS file, using either Tanimoto or Tversky [43] similarity. It supports single query and multiple query searches, as well as the *N* × *N* symmetric case.

The fpcat tool can be used to merge multiple FPS and FPB files together. For example, if the sdf2fps tool is used to extract fingerprints from each file in a PubChem distribution, then fpcat can join them together into a single file.

Version 2.0 of the package added Tversky similarity search and support for the FPB binary file format, as well as support for more than 2 GiB of fingerprint data. Each of the above tools supports reading and writing from FPS, gzip-compressed FPS, and FPB files. Fpcat can convert between all three formats.

The chemfp package also includes a well-documented toolkit API for working with fingerprints. All of the features of the command-line tools are available to user-defined programs, along with APIs to help with web services development and to integrate with NumPy and SciPy. The package is designed to work with static data, which is the usual case in research informatics.

The chemfp project also distributes the chemfp_converters package, which converts between the chemfp formats and the fingerprint formats used by several other packages.

### File scan search

Chemfp supports two similarity search modes: file scan and in-memory. The implementations for both modes expect that there will be few hits relative to the entire data set, so the *O*(*n log k*) performance of a priority queue is effectively *O*(*n*). File scans are only used to search an FPS file. While the FPB file is faster to load and search, the FPS format is a good fit for workflows which do not need the complexity of the FPB format. The FPS format can also be used in streaming contexts, such as piping the output of *sdf2fps* to *simsearch* for a one-off query of fingerprints encoded in the SD file data tags.

A file scan is the default search mode when there is one or a small number of queries. It reads a block of text, and for each line finds the location of the fingerprint and id fields. If the fingerprint passes the similarity test, the score and id are saved, either to a list for a threshold test, or a bounded priority queue for *k*-nearest search.

An important goal when chemfp parses an FPS file is to verify that the file is actually in FPS format, and provide a useful error message if it is not. The exception is that *k*-nearest file scan search terminates early once *k* exact matches are found, instead of verifying the rest of the file.

The secondary goal is to demonstrate that good performance is possible using a text file. A general purpose design might have a file parser which produces a sequence of record objects, and a search algorithm which accepts record objects. This design makes it easy to support multiple file formats by replacing the parser with a new one. However, the intermediate object creation adds unneeded overhead. The similarity can instead be computed directly from the input text. If the score is too low then there’s no need to create a fingerprint record at all, and if the score is high enough then only the identifier and score are needed, not the intermediate fingerprint object.

Chemfp has four different file scan implementations; one for each combination of {Tanimoto, Tversky} × {threshold, *k*-nearest} searches. This level of specialization is less flexible and has a higher development and maintenance cost. On the other hand, if the FPS format becomes the de facto standard fingerprint exchange format then there is less need for a design which can handle multiple formats.

The current file scan implementation processes about 500–600 MiB/s on the benchmark machine. It is not I/O limited as GNU wc 8.25 is able to count newlines in the same file at up to 7 GiB/s. (See Additional file 1: Table S2).

### In-memory search

If there are more than a few tens of queries against an FPS file then it is faster for chemfp to create and search an in-memory data structure called a “fingerprint arena”, which contains four parts. The fingerprints are stored in a contiguous memory block and sorted by population count, such that all fingerprints with 0 bits set come first, followed by those with 1 bit set, and so on. The identifiers are stored in a list in the same order as the fingerprints, such that *id[i]* stores the id for *fingerprint[i]*. The population count index contains a list of fingerprint indices such that the fingerprints with a population count of *b* bits have an index *i* where *popcount_index[b]* ≤ *i* < *popcount_index[b* + *1]*. If no fingerprints have *b* bits set then *popcount_index[b]* = *popcount_index[b* + *1]*. Finally, there is a multi-valued hash table mapping each fingerprint id to 1 or more fingerprint indices. Duplicate ids are allowed.

**Fig. 3:**
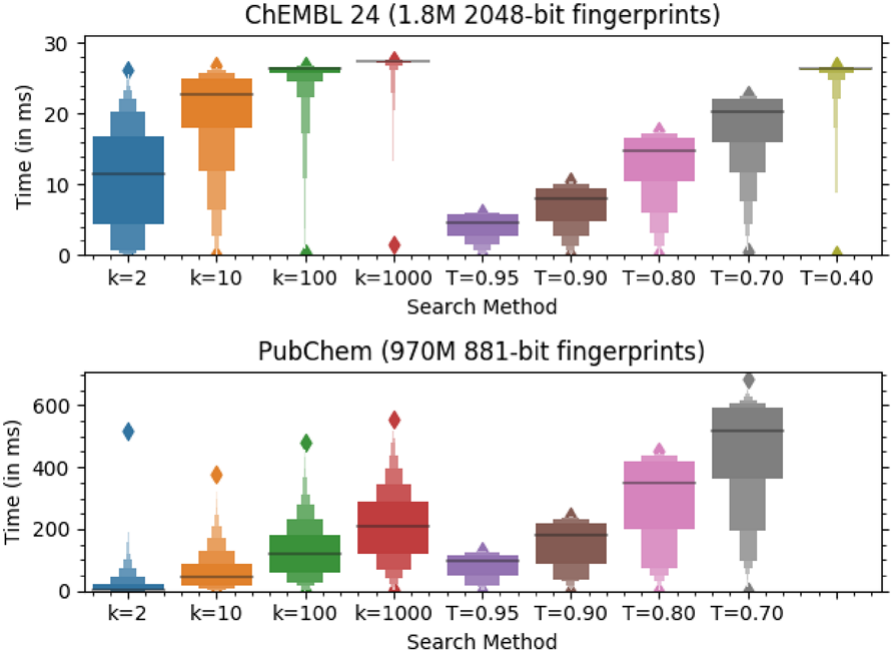
Single query search times for chemfp 3.3. Boxen plots for *k* = 2, 10, 100, and 1000 nearest-neighbor and threshold = 0.95, 0.80, 0.70, and 0.40 searches of ChEMBL 24 and PubChem (downloaded 2018-12-07). Each search samples 1000 fingerprints to use as queries so each query is always found in the result. Python’s garbage collector was disabled for each timing as it adds a roughly 25 ms delay about every 1000 timings. The *T* = 0.40 PubChem search could not be run due to insufficient memory

Each fingerprint is *num_bytes* bytes long and stored in *storage_size* bytes of memory. Zero padding may be added after the fingerprint bytes, typically so the storage size is a multiple of 8 bytes, which lets chemfp use the 64-bit POPCNT instruction. The first fingerprint is located *start_padding* bytes into the memory block, where the offset is chosen so the fingerprints are word or cache-line aligned. Chemfp determines the start padding based on the fingerprint size: 1024-bit fingerprints are 64-byte aligned for possibly better AVX2/AVX512 performance, while 166-bit fingerprints (which are zero padded to 24 bytes), are 8 byte aligned for possibly better POPCNT performance. The distribution of 1000 randomly sampled single query search timings for ChEMBL 24 and PubChem are shown in Fig. 3. The primary reason for the search time variability is because the BitBound pruning effectiveness depends on the population count of the query fingerprint compared to the distribution of fingerprint population counts in the target dataset.

The amount of memory needed for a fingerprint arena is a function of the fingerprint size, alignment, identifier size, and number of records. Only a small amount of additional memory is needed to use a memory-mapped FPB file so see the measured FPB file sizes in Table 3 for approximate memory requirements. Additional memory is required when constructing an arena from an FPS file.

The popcount index of a sorted arena is a compact way to store pre-computed popcounts for all of the target fingerprints, which means that only the intersection popcount is needed for each similarity calculation. The index is also used to apply the BitBound limits. A threshold search only needs to test fingerprints *i* where *popcount_index[floor(b* * *t)]* ≤ *i* < *popcount_index[ceil(B/t)* + *1]*. The implementation uses a minor variation of the *k*-nearest algorithm described in [23]. Instead of sorting an auxiliary array to determine the target popcounts to visit, it does a merge sort of the two monotonically decreasing sides, which can be done in constant memory.

Chemfp also supports unsorted arenas, which are most often used to aggregate multiple small fingerprint sets in a single file. Unsorted searches require a full linear search with both intersection and union popcount calculations.

Chemfp selects the optimal intersection popcount algorithm based on the processor instruction set, fingerprint storage size, and alignment. If the AVX2 instruction set is available, it will be used if the storage size is a multiple of 1024 bits. If the POPCNT instruction is available, it will be used if the storage size is a multiple of 64 bits. Other implementations are available for older hardware and other fingerprint sizes.

The fingerprint size is constant for the entire search, so chemfp implements fully unrolled versions of the AVX2 and POPCNT popcount implementations for fingerprints with a storage size of 24, 64, 112, 128, and 256 bytes. These are used for 166-bit MACCS keys, 512-bit fingerprints, 881-bit PubChem fingerprints, 1024-bit fingerprints, and 2048-bit fingerprints, respectively, when the bits are zero-padded to the next multiple of 64 bits.

The generic threshold and *k*-nearest search methods use a function pointer to call the appropriate intersection popcount implementation. The function call overhead becomes noticeable for high-performance implementations, so there are specialized versions of the AVX2 and POPCNT implementations which inline the popcount calculation. Inlining may also allow the compiler to apply more optimizations. The specialized AVX2 versions only need to initialize the register with the nibble table once, and the versions for exactly 128 and 256 bytes also load the query fingerprint into AVX2 registers only once. The fully unrolled and inlined AVX2 version is the only version which is fast enough for explicit memory prefetching instructions to make a noticeable improvement.

Chemfp does not have special support for handling duplicate fingerprints. If duplicates are removed by an input filter then the k-nearest search becomes a search for the *k*-nearest distinct fingerprints.

Chemfp represents scores as 64-bit floating point values (“doubles”) because it is designed for Python, which uses doubles as the native floating point type. The 64-bit division required for Eq. 3 is relatively slow, so chemfp includes a fast rejection test using integer mathematics on the assumption that most fingerprints will not pass the rejection test. At the start of the search, the input threshold double is converted into a rational number which is equal to or slightly smaller than the threshold value, resulting in a very effective rejection test. An alternative under development is to compute the minimum required popcount given the query and target popcount. Preliminary results suggest a 20% speedup for 166-bit fingerprints but a very minor speedup for 2048-bit fingerprints.

### chemfp benchmark

As Haque et al. [15] highlight, it is difficult to determine if a new search method is effective when the baseline comparison is not well optimized. Authors have an understandable tendency to spend more time optimizing a new algorithm than a seemingly simple linear one, and few have realized that significant gains were possible in linear search.

The authors of some of the published papers commendably also distribute their source code, making a head-to-head comparison possible. However, and again for understandable reasons, many of these are written to demonstrate effectiveness and not as general purpose tools. As specific examples, the implementation might only handle fingerprints which are a multiple of 512 bits long, or require the input files use a specific file-system layout, or report timing information but not the match identifiers and scores.

The chemfp project started with a different goal in mind than most other projects. It distributes general-purpose command-line tools and a library API to help promote the FPS format. The similarity search performance has been improved over time in the expectation that people would use chemfp because of its performance, and thus help popularize the FPS format.

The author of this paper therefore proposes that creators of new methods use the no-cost/open source version of chemfp as a reference for performance comparisons. This should provide a more rigorous baseline, and may be a useful way to normalize timings across multiple papers.

The chemfp benchmark suite takes that idea one step further by providing a collection of fingerprint data sets and tasks which can be used to evaluate search performance.

In all four cases, 1,002,000 fingerprints were sampled at random, without replacement. Of these, 2000 are designated as queries, and the remaining 1 million are targets. Only the first 1000 queries are used during comparison timings. The remaining 1000 queries may be used to double-check the stability of the timings.

The benchmark suite includes a set of standard tasks: count or find all matches at or above a given threshold (0.4 for the Morgan fingerprints and 0.7 for the others), and find the *k*-nearest neighbors (for *k* = 1 and *k* = 1000). The 0.4 threshold task emulates a search to select everything above a background level of similarity for the Morgan similarity. The other threshold levels emulate a more typical search for “good” similarity. These tasks are not meant to be comprehensive, but only to provide an easily interpreted rough estimate of performance.

Table 4 shows the single query search times for chemfp 1.5 and chemfp 3.3, which are the current versions of the no-cost and commercial development tracks. A version of chemfp 1.5 was instrumented to record the number of intersection calculations needed and thereby estimate the effective memory read bandwidth, and to count the number of Tanimoto calculations which required a 64-bit division (see Additional file 1: Table S3). The chemfp 1.5 bandwidth of 11 GiB/s approaches the measured pmbw RAM read bandwidth of 13.3 GiB/s. These numbers show that chemfp 1.5 is likely an effective baseline for similarity search comparisons.

**Table 4:** Average performance of 1000 queries against 1 million targets

#bits	Method	#Tanimotos (M)	chemfp 1.5			chemfp 3.3		
			Avg. time (ms)	T_Tanimoto_ (ns)	Bandwidth (GiB/s)	Avg. time (ms)	T_Tanimoto_ (ns)	Bandwidth (GiB/s)
166	k = 1	91.8	0.25	2.68	8.34	0.19	2.08	10.7
166	k = 1000	588	2.20	3.74	5.97	1.85	3.15	7.10
166	T = 0.70	688	1.72	2.50	8.93	1.42	2.07	10.8
881	k = 1	146	1.50	10.3	10.2	1.22	8.35	12.5
881	k = 1000	485	5.64	11.6	8.97	4.73	9.75	10.7
881	T = 0.70	554	5.70	10.3	10.2	4.70	8.47	12.3
1021	k = 1	113	1.30	11.5	10.4	0.86	7.56	15.8
1021	k = 1000	743	9.25	12.5	9.58	6.25	8.41	14.2
1021	T = 0.70	489	5.51	11.3	10.6	3.64	7.45	16.0
2048	k = 1	356	7.76	21.8	11.0	5.29	14.8	16.1
2048	k = 1000	939	21.2	22.6	10.6	14.6	15.5	15.4
2048	T = 0.40	920	19.9	21.6	11.1	13.6	14.8	16.1

The timings use three different search methods to search the four different fingerprint types from the chemfp benchmark data set. The total number of Tanimoto evaluations is less than 1 billion because of BitBound pruning. T_Tanimoto_ is the average time per Tanimoto evaluation, including storing the hits. The effective read bandwidth is calculated as #Tanimotos * storage_size (24, 112, 128, and 256 bytes respectively)/T_Tanimoto_. Note that while shorter fingerprints are faster and more compact, longer fingerprints tend to have better scientific usefulness

Chemfp 3.3 is faster than chemfp 1.5 because of inlining, AVX2, and the use of explicit memory prefetching instructions. The geometric mean of the ratio of their search times is 1.35 indicating that chemfp 3.3 is about 35% faster than chemfp 1.5. Chemfp 3.3 uses 16 GiB/s of memory bandwidth, which is over half of the theoretical maximum of 25 GiB/s on the test machine and higher than the measured pmbw bandwidth. The current hypothesis is that the chemfp timings include some L3 cache reuse.

### Multiquery searches

The earlier description focused on single query performance. Many common search tasks require multiple queries, such as the *N* × *M* case of comparing two different data sets, or the *N* × *N* case of generating a similarity matrix for clustering, where the fingerprints are used as both queries and targets. In the *N* × *N* case the diagonal is not computed.

Chemfp uses the OpenMP “parallel for” pragma to parallelize each query on its own thread. The *N* × *N* threshold search computes the upper triangle in parallel then uses a single thread to fill in the lower triangle, which roughly doubles the performance. As a minor complication, the OpenMP implementation with one thread was slightly slower than the non-OpenMP version. Chemfp therefore compiles two code paths, and uses the non-OpenMP version for single-threaded use.

Table 5 shows the scalability in the number of processors for different search methods using the 2048 bit data files from the chemfp benchmark. Several of chemfp customers with access to more powerful hardware report successful Taylor–Butina clustering of ~ 3 million fingerprints at a threshold of 0.4 within several hours.

**Table 5:** Multiquery search performance

Method	Query size	1 thread	2 threads		4 threads	
		Time (s)	Time (s)	Scaling	Time (s)	Scaling
k = 1	1000	5.31	3.93	1.35	3.69	1.44
k = 1	Sorted	5.24	3.84	1.36	3.50	1.50
k = 1	N × N	7130 (= 1 h 58 m)	5200 (= 1 h 26 m)	1.37	4640 (= 1 h 17 m)	1.54
k = 1000	1000	14.6	10.5	1.39	9.54	1.53
k = 1000	Sorted	14.5	8.42	1.72	6.30	2.30
k = 1000	N × N	15,300 (= 4 h 14 m)	8040 (= 2 h 13 m)	1.90	4690 (= 1 h 18 m)	3.26
T = 0.90	1000	2.95	2.19	1.35	2.03	1.45
T = 0.90	Sorted	2.92	1.65	1.77	1.04	2.81
T = 0.90	N × N	1890 (= 31 m 34 s)	999 (= 16 m 39 s)	1.90	550 (= 9 m 9 s)	3.45
T = 0.80	1000	5.52	4.09	1.35	3.77	1.46
T = 0.80	Sorted	5.47	2.96	1.85	2.03	2.69
T = 0.80	N × N	3490 (= 58 m 9 s)	1830 (= 30 m 25 s)	1.91	1010 (= 16 m 47 s)	3.46
T = 0.70	1000	8.09	5.95	1.36	5.43	1.49
T = 0.70	Sorted	8.07	4.37	1.85	2.80	2.88
T = 0.70	N × N	4930 (= 1 h 22 m)	2580 (= 42 m 57 s)	1.91	1430 (= 23 m 49 s)	3.45
T = 0.40	1000	13.6	9.99	1.36	8.28	1.64
T = 0.40	Sorted	13.6	7.39	1.83	4.54	2.99
T = 0.40	N × N	7120 (= 1 h 58 m)	3710 (= 1 h 1 m)	1.92	2100 (= 34 m 55 s)	3.40

Time to search the 1 million 2048-bit Morgan fingerprints from the chemfp benchmark data set, for different numbers of threads. A query size of “1000” indicates that the first 1000 benchmark queries were used, “sorted” indicates the same 1000 queries sorted by popcount, and “N × N” generates the full sparse similarity matrix for the 1 million target fingerprints

## Results and discussion

### Popcount performance

Some authors of previous papers on this topic argue that if two different algorithms are implemented by the same people then the resulting timing comparison of the implementations fairly characterizes the relative performance of the algorithms. Table 1 suggests this argument is not very strong. Several earlier papers characterize an 8-bit lookup table as a high performance implementation, but there is a sixfold or larger performance difference compared to a POPCNT-based solution, and an order of magnitude difference compared to an optimized AVX2 implementation. This highlights the need to compare a new algorithm implementation to a well-optimized baseline.

The performance difference makes it difficult to assess the validity of many published papers in this field. A paper might show that the authors’ implementation of a new algorithm is twice as fast as their implementation of the BitBound algorithm when both implementations use a 16-bit lookup table to compute the popcount. Yet if the BitBound implementation were replaced with an AVX2 version, the result may be twice as fast as the new algorithm. It’s tempting to believe that replacing the popcount for the new algorithm would also result in a four-fold speedup, but many of the algorithms perform extra work to avoid a slow popcount calculation. The time for that extra work does not change, reducing the overall speedup, and the extra work might not be as easily optimized as the popcount.

### Floating point issues

Testing of chemfp and fingerprint tools from other vendors shows that certain floating point issues are often overlooked. If the user specifies a threshold of 0.7 + 1E−17 then most systems will include matches with a score of 0.7 because the above number, when input as the full string and converted to a double or 32-bit float, has the same representation as 0.7. A more realistic version of this issue occurs in mixed 32/64-bit systems where the similarity search is implemented using 32-bit floats instead of doubles to reduce memory and improve performance. (32-bit division is significantly faster than 64-bit division). The 32-bit value of 0.7, when converted to a double, is smaller than the 64-bit value of 0.7. If the user specifies a threshold of 0.7 using a double, then a 32-bit system might end up returning values which, when converted to double, are slightly less than the double value of 0.7.

It is also possible for valid matches to be excluded, such as when the 32-bit value for 0.8 is slightly smaller than the 64-bit value used internally for the threshold test. While it is a direct consequence of mixed 32/64-bit systems, and well-known to programmers, it can be quite unexpected for non-programmers when one tool finds, say, 20 more hits than another due to tiny differences in numerical representation.

**Fig. 4:**
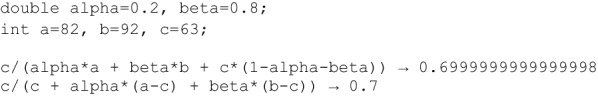
Example of how the non-distributive nature of IEEE 754 doubles results in different Tversky similarity scores

The Tversky similarity is perhaps the second most common similarity measure in cheminformatics, though it is a distant second. The Tversky calculation may cause problems even using homogenous floating point types because IEEE floating point operations do not exactly follow the normal arithmetic distribution rules. Figure 4 shows an example where two seemingly equivalent ways to write the Tversky equation lead to slightly different results. Some values of alpha and beta may even cause the Tversky similarity of a fingerprint with itself to be slightly less than 1.0, which has occurred in both earlier versions of chemfp and other vendor libraries.

Chemfp handles this issue by using integer calculations except for the final division. The values of values of alpha and beta, which are limited to a maximum of 10, are scaled by 10,000 then rounded to the nearest integer. While that introduces a new set of rounding errors, it works because there is no chemically justified reason to have values of alpha and beta with more than two decimal digits of precision.

A likely better design would use scaled decimals and rational values rather than floating point numbers. If the fingerprints have no more than 2^16^ bits then the exact score can be stored in two 16-bit values, with no need for division during search. A post-search step could convert those ratios to 32- or 64-bit floats, as desired. If the Tanimoto threshold is given as a double then it could be converted to a ratio of equal value (which may require 64-bit integers for the numerator and denominator), or replaced by a ratio of two small integers constructed so a search with the new ratio gives the same search results as using the original double.

### Changed cost model

Many papers use an implicit cost model which minimizes the number of intersection popcount calculations and assumes the data structure overhead is small. One of the few explicit cost models in the literature is in Nasr et al. [26], where it was used to optimize a two-stage filtering process.

This model was reasonable 10 years ago, but modern hardware changes one of the simplifications in that approach. The Tanimoto calculation is now fast enough that memory bandwidth and latency are important factors, so the time to compute a Tanimoto is not a constant nor simple distribution, but is a function of the specific data organization and access patterns used.

As an illustrative example, suppose each fingerprint has an associated value like a BitBound popcount or xor signature which is used in a fast filter test to prune obvious mismatches before doing the fingerprint Tanimoto calculation. Chemfp’s AVX2 implementation for 1024-bit fingerprints takes about 7.4 ns per Tanimoto, but if prefetching is disabled it drops to about 8.3 ns. Prefetching cannot be used with a per-fingerprint filter because the fingerprint shouldn’t be fetched until the filter test passes, which is exactly when the fingerprint is needed. In the most optimistic scenario, a filter must therefore be able to remove 10% of the candidates just to break even. A more complete analysis must consider the additional memory bandwidth overhead for the filter values and the higher latency (> 50 ns) of effectively random memory accesses once the fingerprint fetches are no longer easily predictable by the memory subsystem. A similar analysis holds for pruning methods which evaluate a partial fingerprint to determine if the entire fingerprint should be evaluated.

These extra costs can be amortized by grouping fingerprints with the same signature together, which implies either short signatures or a very large set of fingerprints. This conclusion can be derived from a modified version of the Nasr et al. cost model if T_Tanimoto_ is allowed to grow as a function of M and starts with a value closer to T_M=2_.

These issues didn’t arise during the empirical testing in earlier papers because the baseline timings (typically brute-force linear search or a simple test like BitBound) did not come close to reaching bandwidth limitations. Chemfp’s BitBound implementation, for example, is roughly 9× faster than the fastest implementation used in Kristensen et al. [27] for a 0.9 threshold search from the reference benchmark (see Additional file 1: Table S4), in part because the latter depends on the relatively slow performance from representing fingerprints as Java set types. (The TanimotoQuery benchmark was run with OpenJDK 1.8. Additional file 1: Table S5 shows the Kristensen KDGrid is faster than chemfp if the minimum threshold is above 0.97). Nasr et al. describe the time to compute the Tanimoto similarity as 48.8 ± 20.4 μs, which is over 1000× slower than chemfp. That paper uses compressed fingerprints on a machine which did not support the POPCNT instruction.

An alternative is to use deeper tree data structures, but trees tend to have poor data locality, and the effectively random access memory patterns are about an order of magnitude slower than linear access patterns. To be certain, these tree data structures have a finite depth, with linear search in the leaves, so they will be more effective than BitBound for sufficiently large data sets. It’s not clear, however, where that transition occurs.

### Multiquery scaling

Given that a single thread uses about half of the available bandwidth, the prediction is that chemfp’s multiquery search would scale by at most a factor of two, but several benchmarks in Table 5 shows a scaling factor of nearly 3.5 for 4 threads.

The key observation is that the query input order affects the timing. The query sizes of “1000” and “sorted” both use the first 1000 queries from the benchmark data set, though the “sorted” queries are further sorted by popcount. The unsorted queries never scale beyond about 1.6, while the sorted queries often scale beyond 2.0.

The difference can’t simply be due to a more effective use of cache because the single thread time doesn’t change significantly between the sorted and unsorted versions, and because the T = 0.4 search shows good scaling even though the target space is far larger than the ~ 32 K fingerprints which would fit into L3 cache.

The estimate of a maximum of 2× scaling assumes the threads mostly read data from different parts of memory. It appears that some of the search methods have a natural synchronization which causes the threads to read the same memory at nearly the same time. If the query fingerprints are unsorted then the BitBound algorithm causes neighboring threads to read from different fingerprint ranges. If instead the fingerprints are sorted so fingerprints with the same popcount occur together then it’s more likely that many search threads will test the same target fingerprints and in the same order. When one thread is slightly ahead of the other then it will need to wait for the fingerprints to be transferred from RAM, while the slightly slower threads access their data from the significantly faster L3 cache. This temporal coherence explains why the threshold searches, which always examine the same regions of memory for the same popcount, have better scaling than *k*-nearest searches, where the target search space is a function of the *k*th-nearest similarity score. It also explains why the *k* = 1000 searches, which by the law of large numbers will tend to explore the same amount of space, scales better than the more variable reads of *k* = 1 searches.

Some other search tools use more sophisticated methods to handle multiquery searches, for examples by making better use of cache by traversing memory in Morton/Z-order [15] or by query set indexing [44]. These will need to be examined more closely to see if they can be applied to chemfp.

### Benchmark data set density

The 1024-bit and 2048-bit fingerprints in the chemfp benchmark appear to be relatively sparse compared to most path fingerprints for the former, and compared to most fingerprints in general for the second. Density was not considered as part of the selection process because most dense search methods are insensitive to density. However, those choices may affect performance comparisons with sparse methods, which are more sensitive to density.

As a recent paper [45] highlights, the 2048-bit Morgan fingerprints are sparse enough that the RISC algorithm, which uses sparse inverted indices, is faster than chemfp. A closer examination [46] shows that the Morgan fingerprint bit density of only 0.024 for the ChEMBL data set is quite sparse compared to most other 2048-bit fingerprint types. For example, the standard RDKit fingerprint, based on paths and branches up to 7 bonds, has a density of 0.425, and many other fingerprint types have densities above 0.1.

Earlier work [47] showed that clustering 2048-bit Morgan fingerprints using blocked inverted indices was about twice as fast as chemfp 1.1. Together these two papers strongly suggest that sparse methods will outperform dense ones for Morgan fingerprint search—and likely most ECFP-like circular fingerprints. Interestingly, bit position correlations also appear to play a role as RISC and chemfp have comparable performance for a path fingerprint data set with similar sparsity to the Morgan data set.

The Open Babel FP2 fingerprints, which are based on linear fragments of length 1 to 7 atoms, are also unusually sparse for a path fingerprint. Each FP2 hashed path sets only one bit of the fingerprint, while the equivalent fingerprint types from RDKit and OpenEye set more than one bit.

Thus, the chemfp benchmark data sets may not be useful for tasks beyond comparing dense fingerprint methods. On the other hand, while they may not be representative of most fingerprint types, circular fingerprints are widely used, so the benchmark may give more focus to improving their performance. There may also be ways to improve dense approaches to better handle relatively sparse fingerprints. One intriguing possibility is to store compressed fingerprints in memory, and decompress when needed, which has proven useful in other fields when an implementation is memory bandwidth limited [48].

### Funding open source

Starting around 15 years ago a number of papers discussed the role of free and open source software (“FOSS”) in cheminformatics [49–53]. Most papers argued that FOSS was essential for scientific reproducibility and economically beneficial to organizations, but said little about how FOSS projects could be funded, or the effect of the funding model on the project. In practice, most projects are developed through direct research funding or through indirect funding of employees who contribute code to a project. One of the goals of the chemfp project was to explore the possibility of “selling free software” [54] as an alternative funding source. Originally chemfp was only distributed under the MIT license, under the principles of free software. This proved to be financially unsustainable, with low income and poor income stability, so the current distribution also includes cheaper though proprietary licensing options. The rest of this section outlines the issues involved, in hopes of providing insights for future FOSS software projects.

Many FOSS projects are directly funded as part of a research effort. In academic projects, the funding typically comes from grants, and industrial funding typically comes from the R&D budget. The main goal of these projects is the scientific result, and often there is no budget for effective end-user documentation, maintenance, or support, or even portability beyond the developers’ own system. Consequently reported bugs do not get fixed, user questions remain unanswered, and the software often “rots” as it gradually becomes incompatible with evolving software development practices. (There are rare exceptions, as when an academic group is funded as a long-term software resource).

These potential negatives are generally not a problem because most projects are not designed for long-term sustainability. The authors of a journal paper may include an open source implementation as a way for others to verify the result, or a developer may release a package that solved a specific in-house problem as a gift in case others might find it interesting. FOSS software generally doesn’t have the existential issues that a proprietary package may have, in that others can start with the source, though at the cost of rebuilding the lost institutional knowledge of how the software works.

Stability is more important for projects which are deeper in the cheminformatics software stack [55] because users find them more indispensable and harder to maintain independently. The Open Babel, CDK [56], and RDKit toolkits handle this by developing a “community”, that is, an association of people willing to share the labor costs for better stability, more effective impact, esprit de corps, an aversion to proprietary software, a form of apprenticeship, and so on.

These sorts of FOSS projects are generally indirectly funded. One common example is when an employee modifies a project to make it better suited for in-house use, and contributes the modifications back to the primary project maintainers rather than maintain a forked project. The employer indirectly funds the FOSS project, through the employee’s salary, and benefits economically from the exchange. Similar arrangements hold for students and academics. In general, this funding model assumes that participants find a single third party willing to pay them to work on the project. This may be a problem when the needs of the third party are not aligned with the needs of the project, such as when academic researchers find that their career progression is increasingly based on bibliometric counts, and not on leadership or participation in a widely used research software project [57].

Experience across many FOSS projects shows that the community model is nearly always underfunded with respect to the economic and social benefit provided, even for successful projects [58]. For example, a company may decide to use one of the FOSS toolkits instead of paying for a commercial toolkit, but not use any of the savings to help further toolkit development. This is often described as the “free rider problem” [59] of FOSS development, though it is a problem only to the extent that FOSS developers need or expect some sort of compensation. While it’s true that many do not expect monetary payment, many FOSS developers hope for collaborators, contributions of patches and improvements, future consulting work, employment offers, or social or scientific recognition. Even if the developers regard their contribution as a pure gift, it would likely help the project if more of the cost savings from users could be directed towards improving the project. Then again, even if a company wants to contribute funding, it can be hard to figure out how, such as when most of the developers are employees of competing companies.

Many people aren’t even aware that most long-lived, widely deployed FOSS packages—some with millions of users—have only one or a few core developers, and some of these developers get burned out from the emotional stresses involved [58, 60]. Nor is it easy to talk about the need for funding when FOSS development is so closely coupled to terms like “community” and “volunteer” and the software is nearly always available at no cost. Still, these are not completely incompatible topics as some non-software volunteer organizations have paid support staff, as do communities like villages. What are alternative models to pay for FOSS development in cheminformatics?

### Chemfp as commercial open source

Customers will pay for commercial proprietary software, for a price which includes the costs of long-term stability, testing, documentation, and support. Another goal of the chemfp project was to see if industry would pay for commercial FOSS software, where customers who have paid for a copy of the software are then free to use and redistribute it to others, including those who have not paid. Industrial users were the target customer in part because pharmaceutical companies rarely distribute software [53], which reduces some of the economic risk involved.

The initial version of chemfp, which was subsidized by previous consulting income, was not a viable commercial product because it was too slow. It acted as a form of advertising, which lead to several development contracts with companies interested in improved performance, OpenMP parallelism, Tversky search, and the FPB format. Some of these features went into the no-cost version of chemfp, which was meant to promote the use of the FPS format and continue to act as an advertisement for the project, while others went into the commercial version. Both versions were FOSS.

It’s not surprising that the consulting model is one way to fund FOSS development, but its success depends on getting new work. In essence this model views software development as a labor cost, and disregards the capital value of the software. This places it at a disadvantage to commercial software which is able to sell the same software to multiple customers and use the funding to provide additional project support, including the marketing needed to let others know about the project. There is also a perverse incentive in the consulting model because if the software is ‘too good’ there will be less need for consulting [58].

The consulting model doesn’t work well when a lot of work is needed for something which adds little benefit for any one client. Chemfp’s transition from Python 2 to 3 took nearly two months of effort, though to the user the only other difference was support for Unicode identifiers—which are rarely needed. This effort could be justified under the commercial model because the cost was split between customers. An alternative might be the consortium model, where the project doesn’t start until enough people have agreed to pay for it, though that requires additional marketing and sales expertise, and the risk that the effort to build a consortium fails. The consortium model should also be structured to minimize the free rider problem.

The patron model is another variant of the consulting model, where satisfied and supportive users voluntarily contribute to the project, and preferably on a continuing basis. This option was available for chemfp, perhaps designated as a support contract for accounting purposes, though the only contribution so far has been a bottle of wine. (The InChI project uses the patron model as it is funded primarily by membership subscription, though there is also considerable in-kind contributions of time and facilities from its members and other collaborators).

The original chemfp business plan was to use a delayed-release distribution model, where older releases of the commercial version would become the newest release of the no-cost version after, say, 2 years. It was quickly clear that this model would not work because chemfp would be its own competitor. The improvements after only 2 years were not enough to justify a commercial price that could support the entire project development, and many organizations who did not have a pressing need for performance could wait until it was available at no cost.

Instead, chemfp development changed to a two track model. Most new development goes into the commercial track, while the no-cost/open source track is mostly in maintenance mode. This means that chemfp uses a closed development model, while many FOSS projects use an open development model hosted on public servers like GitHub or BitBucket where anyone is free to observe or join. While the closed model may inhibit collaborations with those willing to contribute improvements, personal experience shows that it’s rare for most FOSS projects to get more than occasional patches. The disadvantages of the closed model may easily be outweighed by possible additional funding. For example, funding from chemfp sales was used to pay two people from the small community of popcount optimizers to improve chemfp performance.

### Problems selling free software

It may seem like a contradiction to “sell” free software, because one definition of “free” means available at no cost. There are differences between the social movement of free software and the development methodology of open source [61]. To many they are like the doctrinaire differences between the People’s Front of Judea and the splitters of the Judean People’s Front [62]. For purposes of this paper, the Four Freedoms [63] of free software say that anyone who receives any software has a right to usable source code of that software and is free to use, modify, or redistribute the software and source—including for a fee—so long as the software remains free software. The open source methodology argues that useable source code with few restrictions is better than proprietary software because it results in better software, but it can be acceptable (if the license allows) to include open source software in a binary-only package, or in software which prohibits redistribution.

For many years chemfp followed the free software principles and only distributed under the MIT license, along with some third-party components under an equally permissive license. These principles made it much more difficult to sell chemfp. If a potential customer wants to evaluate the software before buying it, and the evaluation software is distributed under a free software license, then there is little other than a loss of good will which prevents the customer from continuing to use the software but not going through with the sale.

Commercial proprietary software uses market segmentation so that the sale price is a better match for what customers are willing to pay. It is common practice for academic groups to receive a copy of commercial proprietary software at no cost or a greatly reduced cost. However, while a developer at a large pharmaceutical is unlikely to redistribute software, a graduate student at a university is much more likely to make a copy of open source software available to the public. The price for an academic purchase, after factoring in the economic risk that the software may be redistributed, may be more expensive than an industrial purchase. For chemfp this meant that the commercial FOSS version was only sold to industrial customers.

These and similar problems lead to the conclusion that it is not possible to develop chemfp as a self-funded fully free software project. The latest business model assumes that people are mostly interested in FOSS because it is available for no cost, and not because of moral principles or an improved development methodology. All versions of chemfp are still available under the MIT license. What’s new are proprietary licensing options for those who do not wish to pay the full price, and pre-compiled binaries with a time-locked license key for evaluation purposes. The change to include proprietary licensing was only possible because chemfp does not depend on any components under a free software license like the GPL which requires that derived distributions always be free software.

This does not mean that self-supported commercial FOSS software for cheminformatics is impossible. There might not be enough demand for chemfp, it may have the wrong pricing model or insufficient marketing, or any of the many reasons which cause a product to fail.

### Open core

Chemfp is one of perhaps a handful of self-funded FOSS projects in cheminformatics or related fields. The best known was the early commercialization of PyMol [64], which sought voluntary contributions from users. As an incentive, those who paid could download pre-compiled binaries (anyone could download and compile the source) and have access to features that were not generally available. PyMol was a very successful project in terms of the number of users, but DeLano Scientific never received enough funding to hire another full-time employee, and augmented its income by providing consulting services to produce publication quality structure images [Warren DeLano, personal communication]. Chemfp was influenced by PyMol’s model, though focusing on corporate sales and not end-users or academics.

Many more successful FOSS projects instead follow what is broadly referred to as “open core” [65] where one product is available as FOSS, as part of a larger suite which includes proprietary software. The proprietary software may be a more advanced version of the product or plug-in extensions, or there may be easy integration between the FOSS product and other vendor products. From a business viewpoint, the FOSS product can be justified as a loss leader or as advertising for the proprietary products which fund development. Overall, open core seems the most successful alternative funding mechanism for developing FOSS products.

However, this approach requires giving up on the ideals of free software and coming up with multiple successful products. Since the core is likely funded with the expectation of future income from a proprietary product, there is also the economic risk that others will use the open core component to develop a competitor to the proprietary version but without taking on the development debt.

### Status and future

Chemfp is used at many companies, though most use the no-cost version. One of the more unusual examples is when one company used it in a comparison between two proprietary corporate compound libraries [66]. Chemfp’s performance was fast enough that a full nearest-neighbor analysis could be done in less than a day on an isolated laptop; the hard drive was then reformatted after the analysis to preserve confidentiality [Thiery Kogej, private communication].

It is hard to get a sense of who uses the no-cost version of chemfp or the FPS format, in part because modern FOSS distribution is increasingly intermediated by package managers. There are about 20 anonymous downloads per month from the chemfp web server, and another 15 through BioConda. (The BioConda distribution is not maintained by the chemfp project). It’s likely that most people download chemfp through PyPI, the Python Package Index, which uses its own copy of the source and does not provide download statistics. A download may correspond to one user, or to a system administrator who installs it for an entire organization, or a continuous integration system which repeatedly downloads the package.

Informal conversations at conferences suggest that chemfp is well-known and widely deployed, but the FPS format has not started to replace the ad hoc internal formats that it was designed to replace.

Several packages do support the FPS format, including CACTVS [67], Open Babel, and the ‘fingerprint’ package for R [68]. ChEMBL includes pre-computed RDKit Morgan fingerprints in FPS format as part of the standard distribution, starting with version 24 from March 2018. The FPB format was not publicly documented until 2018. At present only RDKit and the commercial version of chemfp support it. The chemfp benchmark has been used to evaluate the RISC algorithm, and its implementation also supports the FPS format.

While chemfp handles most of the common similarity search tasks in cheminformatics, there is still much that can be done, like a diversity picker, or plugin support to make it easier to add new fingerprint types. Some analysis methods, like generating a histogram of all N × M similarities between two large data sets, are a natural fit for Morton/Z-ordering and seem like an excellent candidate for future inclusion in chemfp.

Computing hardware continues to improve. Chemfp will add support for the 512-bit VPOPCNTDQ instruction, which should be a good complement to the higher bandwidth of DDR4 memory. Future research will likely evaluate the effectiveness of other pruning methods, with particular attention on M = 2 pruning. This research will also inform the changes to the FPB format which are needed to effectively support real-world data sets, which are approaching 1 billion compounds.

GPU memory bandwidth is an order of magnitude higher than CPU bandwidth, so a GPU implementation of the Tanimoto search kernel should be about ten times faster. Chemfp has avoided GPU support so far because it’s not clear that the demand for similarity search justifies dedicated hardware, especially if the time to load the data into the GPU is slower than the time to search it on the CPU. GPUs are more likely to be appropriate for clustering mid-sized datasets where the fingerprints fit into GPU memory.

Those are mostly engineering topics. One of the more interesting scientific topics in chemfp’s long-term future is support for sparse fingerprints and sparse count fingerprints. Dense fingerprints are often created by folding a sparse fingerprint (typically with a few hundred bits set in the range 2^32^ or 2^64^) down to 1024 or 2048 bits. Some authors suggest that significantly larger folded fingerprints—as long as 16,384 bits—are more appropriate for some tasks [69]. While chemfp can handle these longer fingerprints, the increased length requires more memory and decreases search performance.

It seems that a better solution would be to develop tools designed to work directly on sparse count fingerprints. While such tools already exist, they are not widely used. Informal discussions suggest that people aren’t using them because the tools don’t have anywhere near the same performance or level of maturity as dense fingerprint search tools, which makes it harder to gain the experience to judge when sparse count fingerprints are useful, which in turn reduces the push to improve the tools. This is another chicken-and-egg problem which seems a natural fit for the chemfp project.

## Conclusions

Tanimoto similarity search on modern hardware is essentially limited by memory bandwidth, which means a rough estimate of the maximum search time is roughly the number of fingerprints times the number of bits divided by the RAM bandwidth. Further improvements are possible by pruning the search space, and there many publications along these lines. Few of those papers used a Tanimoto calculation implementation which approaches the bandwidth limits, in part because popcount evaluation on older hardware was not fast enough, and in part because many implementations did not use the fastest available methods.

That does not mean these more sophisticated methods are invalid. Instead, it shows how difficult is is to compare two algorithms through specific implementations. Some algorithms are a better fit to the hardware, and two people of the same skill may produce implementations of the same algorithm with a several-fold difference in performance. Empirical testing shows that chemfp’s basic BitBound implementation is around nine times faster than an implementation of MultibitTree. It may be that a more optimized version of MultibitTree is even faster; at present we don’t know.

Chemfp is not the first program to approach memory bandwidth bounds, but it is the first one available as a general-purpose toolkit. The no-cost, MIT-licensed version, while about 25–35% slower than the commercial version, should provide a useful reference baseline for new work, and the chemfp benchmark should make it easier to do head-to-head comparisons. Using chemfp in this way will also promote the FPS format, which is the main reason for starting the chemfp project.

Chemfp was organized as commercial FOSS project, to experiment with an alternative way to fund FOSS software development. It was not financially successful so proprietary licensing models were added. While some of the difficulties are specific to chemfp, others will be true for any commercial project with a pure FOSS business model. It’s still unclear how FOSS can be funded in a way that reflects its importance to a large number of users. Without some sort of funding mechanism, the only people who will be able to work on FOSS projects are those who can convince their employer that it is worthwhile, and those who do it as a hobby. This is unlikely to scale as more and more people use FOSS in cheminformatics. We already see how the lack of funding has lead to problems in the larger world of FOSS. The author hopes others are able to come up with a better solution for our field.

## Supplementary information

**Additional file 1: Table S1.** Performance of different popcount implementations, in milliseconds and relative to the 8-bit lookup table time, measured using the threshold searches from the chemfp benchmark suite (*T* = 0.4 for 2048 bit searches, otherwise *T* = 0.7). In most cases the search algorithm uses a function pointer to dispatch to the appropriate popcount function, without memory prefetching. The POPCNT and AVX2 versions show times using loops of different sizes and “fully unrolled” versions which implement the fingerprint popcount without a loop. The ‘inline’ and ‘prefetch’ variants inline the calculation and use memory prefetching, respectively. Timings were made with chemfp 3.3. **Figure S1.** Scaling of *k* = 1 nearest neighbor searches as a function of the number of targets, for different fingerprint types. MACCS and FP2 fingerprints scales as *O*(*n*^~0.65^) and the PubChem/CACTVS and Morgan searches scale as *O*(*n*^~0.8^) in the number of fingerprints in the dataset, which is the sublinear scaling expected from using BitBound. Timings made with chemfp 1.5. **Table S2.** Chemfp file scan search performance for 100 queries from each of the data sets in the chemfp benchmark. The search time shows chemfp processes 500–600 MiB/s. The GNU program “wc” version 8.25 can count the number of lines in about 1/10th the time indicating that chemfp is not disk I/O bound. **Table S3.** Number of Tanimotos evaluated for an in-memory search of each of the test cases in the chemfp benchmark suite. The number of Tanimotos is much less than the expected 1 billion (1000 queries * 1 million targets) because of the BitBound limits. The number of divisions is the number of tests which passed the fast rational rejection test so require a 64-bit division. It shows the effectiveness of the rational rejection test. **Table S4.** Performance comparison as a function of the number of fingerprints between the fastest implementation from Kristensen et al. [28] and chemfp 3.3, using the Kristensen benchmark data set. The benchmark does a threshold = 0.9 search using the first 100 fingerprints in the data set. **Table S5.** Performance comparison as a function of minimum Tanimoto threshold between the fastest implementation from Kristensen et al. and chemfp 3.3, using the Kristensen benchmark data set. The benchmark uses the first 100 fingerprints in the data set to search the first 1,999,998 fingerprints. LinearSearcher is the fastest Kristensen method for all Tanimoto thresholds at or below 0.76. Some thresholds timings are omitted here as they add little useful information. The full table for each threshold step of 0.01 is available from this paper’s BitBucket repository.

## Data Availability

Both the no-cost and commercial versions of chemfp are available from http://www.chemfp.com/ as well as the FPS and FPB format specifications. A no-cost license is available from the author on reasonable request for those wishing to verify the results of this paper. The chemfp benchmark is available from https://bitbucket.org/dalke/chemfp_benchmark. The data sets generated during the current study, along with code used to produce and analyze them, are available at https://bitbucket.org/dalke/chemfp_paper_reproducibles/.

## Abbreviation

FOSS: free and open source software
