# Correction to: The chemfp project

**DOI:** 10.1186/s13321-020-00459-y

**Published:** 2020-09-28

**Authors:** Andrew Dalke

**Affiliations:** Andrew Dalke Scientifc AB, Trollhättan, Sweden

## Correction to: J Cheminform (2019) 11:76 10.1186/s13321-019-0398-8

It was highlighted that the original article [1] contained a mistake in the Abstract and in the legend of Fig. 3. This Correction article shows the incorrect and correct sentence of the Abstract and the correct Fig. 3.

Incorrect sentence

The same search of 970 million PubChem fingerprints averages 220 ms/query, making chemfp one of the fastest CPU-based similarity search implementations.

Correct sentence

The same search of 97 million PubChem fingerprints averages 220 ms/ query, making chemfp one of the fastest CPU-based similarity search implementations.

**Fig. 3:**
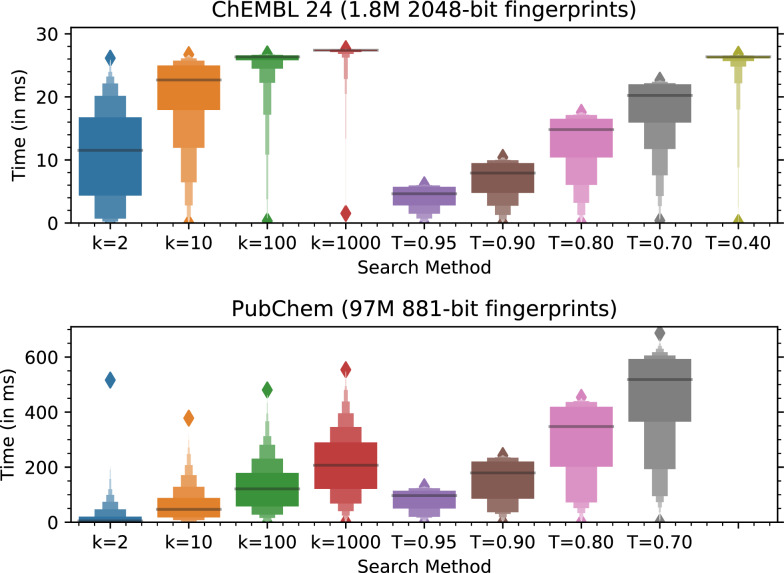
The same search of 97 million PubChem fingerprints averages 220 ms/query, making chemfp one of the fastest CPU-based similarity search implementations
