# Supplementary material for: The chemfp project
Source: J Cheminform. 2019 Dec 5;11:76. doi: 10.1186/s13321-019-0398-8 (PMC6896769; doi:10.1186/s13321-019-0398-8)
Supplement: Supplementary file 1 — Additional file 1: Table S1. Performance of different popcount implementations, in milliseconds and relative to the 8-bit lookup table time, measured using the threshold searches from the chemfp benchmark suite (T = 0.4 for 2048 bit searches, otherwise T = 0.7). In most cases the search algorithm uses a function pointer to dispatch to the appropriate popcount function, without memory prefetching. The POPCNT and AVX2 versions show times using loops of different sizes and “fully unrolled” versions which implement the fingerprint popcount without a loop. The ‘inline’ and ‘prefetch’ variants inline the calculation and use memory prefetching, respectively. Timings were made with chemfp 3.3. Figure S1. Scaling of k = 1 nearest neighbor searches as a function of the number of targets, for different fingerprint types. MACCS and FP2 fingerprints scales as O(n~0.65) and the PubChem/CACTVS and Morgan searches scale as O(n~0.8) in the number of fingerprints in the dataset, which is the sublinear scaling expected from using BitBound. Timings made with chemfp 1.5. Table S2. Chemfp file scan search performance for 100 queries from each of the data sets in the chemfp benchmark. The search time shows chemfp processes 500–600 MiB/s. The GNU program “wc” version 8.25 can count the number of lines in about 1/10th the time indicating that chemfp is not disk I/O bound. Table S3. Number of Tanimotos evaluated for an in-memory search of each of the test cases in the chemfp benchmark suite. The number of Tanimotos is much less than the expected 1 billion (1000 queries * 1 million targets) because of the BitBound limits. The number of divisions is the number of tests which passed the fast rational rejection test so require a 64-bit division. It shows the effectiveness of the rational rejection test. Table S4. Performance comparison as a function of the number of fingerprints between the fastest implementation from Kristensen et al. [28] and chemfp 3.3, using the Kristensen benchmark data set [file 13321_2019_398_MOESM1_ESM.pdf]

Supplementary Data for

“The chemfp Project”

Andrew Dalke <dalke@dalkescientific.com>

Andrew Dalke Scientific AB, Trollhättan, Sweden

	Performance measured in milliseconds (scaled to 8-bit LUT)			
**Popcount method**	**166 bits**	**881 bits**	**1024 bits**	**2048 bits**
8-bit lookup table	9.41 (1.0x)	38.6 (1.0x)	39.7 (1.0x)	144 (1.0x)
16-bit lookup table	4.81 (2.0x)	13.6 (2.8x)	13.5 (2.9x)	60.1 (2.4x)
Gillies-Miller	5.96 (1.6x)	13.2 (2.9x)	12.7 (3.1x)	41.9 (3.4x)
Lauradoux		12.4 (3.1x)	12.2 (3.3x)	39.3 (3.7x)
SSSE3			7.40 (5.4x)	23.8 (6.1x)
POPCNT (8 bytes/loop)				
dispatch	2.60 (3.6x)	6.43 (6.0x)	6.29 (6.3x)	22.4 (6.4x)
inline	1.91 (4.9x)	5.89 (6.6x)	5.79 (6.9x)	21.7 (6.6x)
POPCNT (32 bytes/loop)				
dispatch			6.29 (6.3x)	22.5 (6.4x)
inline			5.81 (6.8x)	21.7 (6.6x)
POPCNT (128 bytes/loop)				
dispatch			5.49 (7.2x)	18.6 (7.7x)
inline			5.47 (7.3x)	18.6 (7.7x)
POPCNT (fully unrolled)				
dispatch	1.76 (5.3x)	4.91 (7.9x)	4.84 (8.2x)	18.5 (7.8x)
inline	1.41 (6.7x)	4.71 (8.2x)	4.72 (8.4x)	18.1 (8.0x)
AVX2 (128 bytes/loop)				
dispatch			4.62 (8.6x)	15.7 (9.2x)
dispatch,prefetch			4.60 (8.6x)	15.5 (9.3x)
inline			4.61 (8.6x)	15.7 (9.2x)
inline, prefetch			4.58 (8.7x)	15.6 (9.2x)
AVX2 (fully unrolled)				
dispatch			4.60 (8.6x)	15.6 (9.2x)
dispatch, prefetch			4.57 (8.7x)	15.5 (9.3x)
inline			4.04 (9.8x)	14.5 (9.9x)
inline, prefetch			3.61 (11.0x)	13.5 (10.6x)

Table S1: Performance of different popcount implementations, in milliseconds and relative to the 8-bit lookup table time, measured using the threshold searches from the chemfp benchmark suite (T=0.4 for 2048 bit searches, otherwise T=0.7). In most cases the search algorithm uses a function pointer to dispatch to the appropriate popcount function, without memory prefetching. The POPCNT and AVX2 versions show times using loops of different sizes and "fully unrolled" versions which implement the fingerprint popcount without a loop. The 'inline' and 'prefetch' variants inline the calculation and use memory prefetching, respectively. Timings were made with chemfp 3.3.

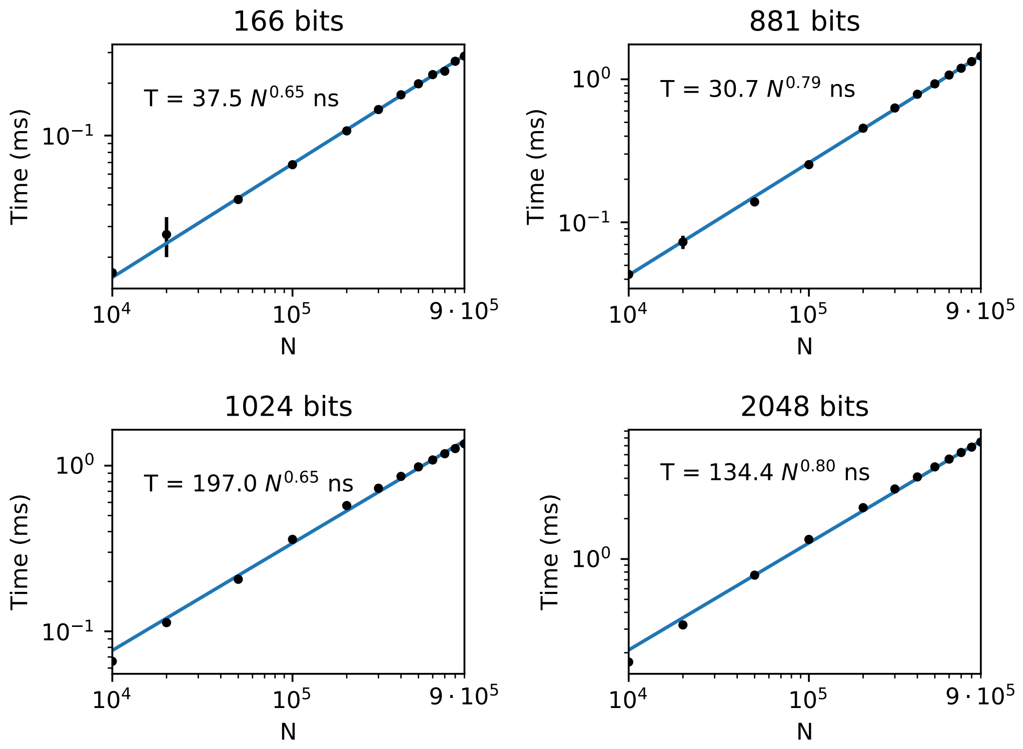

Figure S1: Scaling of k=1 nearest neighbor searches as a function of the number of targets, for different fingerprint types. MACCS and FP2 fingerprints scales as O($n^{\sim 0.65}$) and the PubChem/CACTVS and Morgan searches scale as O($n^{\sim 0.8}$) in the number of fingerprints in the dataset, which is the sublinear scaling expected from using BitBound. Timings made with chemfp 1.5.

# Bits	Method	Average time (in s)	Minimum time (in s)	Maximum time (in s)	Bandwith (in MiB/s)
166	wc	0.014	0.014	0.015	3848.1
166	k=1	0.10	0.07	0.12	522.7
166	k=1000	0.12	0.12	0.12	466.9
166	T=0.7	0.12	0.12	0.14	444.3
881	wc	0.037	0.036	0.038	6040.3
881	k=1	0.41	0.41	0.42	538.8
881	k=1000	0.41	0.41	0.42	535.2
881	T=0.7	0.44	0.43	0.46	509.0
1024	wc	0.041	0.041	0.044	6224.3
1024	k=1	0.43	0.22	0.48	594.2
1024	k=1000	0.47	0.47	0.47	547.9
1024	T=0.7	0.49	0.49	0.51	526.0
2048	wc	0.072	0.071	0.074	6972.0
2048	k=1	0.84	0.38	0.89	597.0
2048	k=1000	0.86	0.86	0.87	582.1
2048	T=0.4	0.90	0.89	0.94	555.8

Table S2: Chemfp file scan search performance for 100 queries from each of the data sets in the chemfp benchmark. The search time shows chemfp processes 500-600 MiB/s. The GNU program “wc” version 8.25 can count the number of lines in about 1/10th the time indicating that chemfp is not disk I/O bound.

# Bits	Method	# Tanimotos	# Divisions
166	k=1	91825922	17476
166	k=1000	587628687	7267986
166	T=0.7	687772183	5632389
881	k=1	146240123	13683
881	k=1000	485142743	6447790
881	T=0.7	554424332	6392581
1024	k=1	113231403	13043
1024	k=1000	742888893	7024476
1024	T=0.7	488805676	314319
2048	k=1	356438671	15154
2048	k=1000	939409454	7502635
2048	T=0.4	920844855	393145

*Table S3: Number of Tanimotos evaluated for an in-memory search of each of the test cases in the chemfp benchmark suite. The number of Tanimotos is much less than the expected 1 billion (1,000 queries * 1 million targets) because of the BitBound limits. The number of divisions is the number of tests which passed the fast rational rejection test so require a 64-bit division. It shows the effectiveness of the rational rejection test.*

# Fingerprints	Fastest method from Kristensen *et al.*	Average search time (in ms)	chemfp time (in ms)	Ratio
99999	KDGrid-2-ListBucket	1.58	0.12	13.17
199999	KDGrid-1-MultibitTree	3.16	0.25	12.69
299999	KDGrid-1-MultibitTree	4.85	0.41	11.89
399999	KDGrid-1-MultibitTree	6.28	0.58	10.75
499999	KDGrid-1-MultibitTree	7.67	0.76	10.12
599999	KDGrid-1-MultibitTree	9.13	0.93	9.87
699999	KDGrid-1-MultibitTree	10.60	1.10	9.67
799999	KDGrid-1-MultibitTree	11.73	1.24	9.47
899999	KDGrid-1-MultibitTree	13.28	1.41	9.40
999999	KDGrid-1-MultibitTree	14.47	1.57	9.21
1099999	KDGrid-1-MultibitTree	15.59	1.72	9.09
1199999	KDGrid-1-MultibitTree	16.97	1.86	9.12
1299999	KDGrid-1-MultibitTree	17.28	2.02	8.56
1399999	KDGrid-1-MultibitTree	18.44	2.25	8.19
1499999	KDGrid-1-MultibitTree	19.46	2.33	8.35
1599998	KDGrid-1-MultibitTree	21.46	2.48	8.64
1699998	KDGrid-1-MultibitTree	22.10	2.73	8.10
1799998	KDGrid-1-MultibitTree	22.73	2.87	7.91
1899998	KDGrid-1-MultibitTree	24.39	3.01	8.10
1999998	KDGrid-1-MultibitTree	24.91	3.08	8.09

Table S4: Performance comparison as a function of the number of fingerprints between the fastest implementation from Kristensen et al. [28] and chemfp 3.3, using the Kristensen benchmark data set. The benchmark does a threshold=0.9 search using the first 100 fingerprints in the data set.

Threshold	Fastest method from Kristensen *et al*.	Average search time (in ms)	chemfp time (in ms)	Ratio
0.00	LinearSearcher	188.03	41.77	4.50
0.10	LinearSearcher	189.79	33.34	5.69
0.20	LinearSearcher	189.12	31.37	6.03
0.30	LinearSearcher	183.54	19.97	9.19
0.40	LinearSearcher	173.50	13.95	12.44
0.50	LinearSearcher	172.78	12.65	13.66
0.60	LinearSearcher	172.60	10.96	15.74
0.70	LinearSearcher	171.84	8.67	19.82
0.76	LinearSearcher	166.32	7.06	23.57
0.77	KDGrid-1-MultibitTree	162.50	6.78	23.99
0.80	KDGrid-1-MultibitTree	118.11	5.91	19.98
0.85	KDGrid-1-MultibitTree	59.92	4.46	13.45
0.90	KDGrid-1-MultibitTree	23.77	2.97	8.01
0.91	KDGrid-1-MultibitTree	18.96	2.68	7.07
0.92	KDGrid-1-MultibitTree	14.60	2.38	6.13
0.93	KDGrid-1-MultibitTree	10.92	2.09	5.22
0.94	KDGrid-1-MultibitTree	7.78	1.80	4.32
0.95	KDGrid-2-MultibitTree	4.65	1.51	3.09
0.96	KDGrid-2-MultibitTree	2.44	1.22	2.00
0.97	KDGrid-4-ListBucket	0.95	0.94	1.01
0.98	KDGrid-4-ListBucket	0.24	0.66	0.37
0.99	KDGrid-4-ListBucket	0.02	0.37	0.05
1.00	KDGrid-1-ListBucket	0.00	0.05	0.00

Table S5: Performance comparison as a function of minimum Tanimoto threshold between the fastest implementation from Kristensen et al. and chemfp 3.3, using the Kristensen benchmark data set. The benchmark uses the first 100 fingerprints in the data set to search the first 1,999,998 fingerprints. LinearSearcher is the fastest Kristensen method for all Tanimoto thresholds at or below 0.76. Some thresholds timings are omitted here as they add little useful information. The full table for each threshold step of 0.01 is available from this paper's BitBucket repository.
